# Uncontrolled asthma: assessing quality of life and productivity of children and their caregivers using a cross-sectional Internet-based survey

**DOI:** 10.1186/1477-7525-8-96

**Published:** 2010-09-08

**Authors:** Bonnie B Dean, Brian C Calimlim, Patricia Sacco, Daniel Aguilar, Robert Maykut, David Tinkelman

**Affiliations:** 1 Cerner LifeSciences, Beverly Hills, CA, USA; 2 Global Health Economics and Outcomes Research, Novartis Pharmaceuticals Corporation, East Hanover, NJ, USA; 3 US Clinical Development & Medical Affairs, Novartis Pharmaceuticals Corporation, East Hanover, NJ, USA; 4 Department of Pediatrics, National Jewish Medical and Research Center, Denver, CO, USA

## Abstract

**Background:**

Results of a national survey of asthmatic children that evaluated management goals established in 2004 by the National Asthma Education and Prevention Program (NAEPP) indicated that asthma symptom control fell short on nearly every goal.

**Methods:**

An Internet-based survey was administered to adult caregivers of children aged 6-12 years with moderate to severe asthma. Asthma was categorized as uncontrolled when the caregiver reported pre-specified criteria for daytime symptoms, nighttime awakening, activity limitation, or rescue medication based on the NAEPP guidelines. Children's health-related quality of life (HRQOL) and caregivers' quality of life (QOL) were assessed using the Child Health Questionnaire Parent Form 28 (CHQ-PF28) and caregiver's work productivity using a modified Work Productivity and Activity Impairment Questionnaire. Children with uncontrolled vs. controlled asthma were compared.

**Results:**

360 caregivers of children with uncontrolled asthma and 113 of children with controlled asthma completed the survey. Children with uncontrolled asthma had significantly lower CHQ-PF28 physical (mean 38.1 vs 49.8, uncontrolled vs controlled, respectively) and psychosocial (48.2 vs 53.8) summary measure scores. They were more likely to miss school (5.5 vs 2.2 days), arrive late or leave early (26.7 vs 7.1%), miss school-related activities (40.6 vs 6.2%), use a rescue inhaler at school (64.2 vs 31.0%), and visit the health office or school nurse (22.5 vs 8.8%). Caregivers of children with uncontrolled asthma reported significantly greater work and activity impairment and lower QOL for emotional, time-related and family activities.

**Conclusions:**

Poorly controlled asthma symptoms impair HRQOL of children, QOL of their caregivers, and productivity of both. Proper treatment and management to improve symptom control may reduce humanistic and economic burdens on asthmatic children and their caregivers.

## Background

In 2006 there were approximately 6.8 million children 17 years of age or younger with asthma in the United States [1]. Nearly half of these children (46.8%; 3.2 million) were 5-11 years old. With asthma being the third-ranked cause of hospitalization among children younger than 15 years of age [2,3] and the leading cause among children 3-12 years old [2,3], achieving adequate control of asthma symptoms is imperative. Asthma has accounted for more than 14 million school days missed each year and has been linked to diminished school performance [3-5]. It is the most common cause of school absenteeism due to a chronic disease [6]. A decrease in the child's health-related quality of life (HRQOL) and increase in absenteeism may also affect the quality of life (QOL) and work productivity of the child's caregiver, who may lose time from work, change to part-time employment, or choose to not work at all to care for the child.

The last decade has seen a shift in the management of asthma in clinical practice. Rather than managing patients based on their severity, current clinical practice guidelines emphasize that the overall goal of management is to achieve symptom control [7]. Good asthma control has been shown to be associated with improved health status [8]. The importance of symptom control in children is underscored by the results of a national survey of asthmatic children that evaluated asthma management goals established by the National Asthma Education and Prevention Program (NAEPP) in 2004 [9]. The survey found that asthma control fell short on nearly every goal, indicating the lack of effective asthma symptom control in children.

This study was conducted to evaluate the impact of asthma symptom control upon the HRQOL of asthmatic children, the QOL of the children's caregivers, and the productivity of the children with asthma as well as their caregivers. We hypothesized that: 1) children whose asthma disease state was not well controlled have a decreased QOL and lower school productivity compared to children with controlled asthma, and that 2) caregivers of children whose asthma was not well controlled have a decreased QOL and lower work productivity compared to caregivers of children with controlled asthma.

## Methods

### Study Design and Data Source

A random sample from a general registry of Internet users who represented the United States (US) adult population in terms of age, gender, geographic location, and ethnicity was drawn in July 2007. This study was approved by the Western Institutional Review Board (WIRB). Prior to completing the survey, all respondents were required to review and provide individual "sign-off" on an IRB-approved electronic consent form, which provided a brief background on the study, objectives and risks of participation. Respondents also received toll-free telephone numbers in case they needed to contact the survey provider and/or the WIRB.

Participants were enrolled through e-mail invitations sent by the registry management to households prescreened for registry participation. Invitations were sent to adults in households with at least one child younger than the age of 18. The invitations asked adults to participate in a cross-sectional Internet-based survey of caregivers of asthmatic children aged 6-12. No attempt was made to enlist participants from any particular demographic group or from those under the care of primary care physicians or specialists. The following criteria needed to be met by caregivers who opted to participate in the survey and their children, respectively: the caregiver had to be at least 18 years of age and living in the US, and the caregiver's child was required to be from 6 to 12 years of age, have a doctor's diagnosis of asthma, and have met predefined criteria for moderate to severe asthma (asthma severity is defined below). If more than one child qualified for the study, only the youngest child meeting all the study criteria was included. Participants received points for participation that could be redeemed for items amounting to less than $5.

### Asthma Severity

While many definitions of asthma severity have been developed, none are consistently used, especially within cross-sectional research. Assigning asthma severity in observational studies is also complicated by the level of symptom control achieved through controller medications. Even a patient without daily symptoms could experience episodes severe enough to warrant an indication of moderate to high severity.

A pre-specified algorithm based on the child's healthcare utilization and current medications was used to identify children with moderate to severe asthma. To create this algorithm, caregiver-reported recent medication history was mapped to NAEPP 2002 medication recommendations for the lowest treatment level required to maintain symptom control [10]. Children were classified as having moderate to severe asthma if their caregiver reported ANY of the following criteria: (a) an asthma-related hospitalization within the last year; (b) an intensive care unit admission for asthma-related symptoms within the last year; (c) the child being placed on a ventilator during the last year; (d) daily oral corticosteroid use; (e) daily inhaled corticosteroid use at moderate to high doses according to NAEPP 2002 medication recommendations; or (f) daily use of low-dose inhaled corticosteroids along with any of the following medications: theophylline, leukotriene receptor antagonist, cromolyn, or a long-acting bronchodilator.

### Asthma Symptom Control

Prior to the recent NAEPP guidelines, no clearly defined method was published for assigning symptom control in cross-sectional studies. The current guidelines provide five criteria for assessing symptom control in asthma patients. In this study, symptom control was determined by question responses regarding four of the five key symptom control expressions described in the NAEPP 2007 asthma guidelines [11]: prevention of daytime symptoms, reduction of nocturnal awakening, infrequent short-acting beta agonist use, and participation in normal activity levels. Forced expiratory volume in one second is an office- or hospital-based measure rather than a symptom measure and thus was not collected in this cross-sectional study.

Children were classified as having uncontrolled asthma if their caregiver reported ANY one of the following criteria: (a) symptoms > 2 days per week; (b) awakened by symptoms any night during the past 4 weeks; (c) any activity limitation (in kind or amount) due to impairment or health problem; or (d) rescue inhaler use > 5 times per week. All other children were classified as having controlled asthma.

### Health-related Quality of Life

The Child Health Questionnaire Parent Form 28 (CHQ-PF28) was used to measure the HRQOL of the child with asthma and the QOL of the child's caregiver [12]. A generic HRQOL instrument, the CHQ-PF28 is designed to measure the HRQOL of children and the QOL of their families across 13 scales. The following nine scales measure the child's HRQOL: physical functioning (PF), role/social limitations-emotional/behavioral (REB), role/social limitations-physical (RP), bodily pain/discomfort (BP), behavior (BE), mental health (MH), self-esteem (SE), general health (GH), and change in health (CH). These scales are summarized into a physical summary measure (PHS) and a psychosocial summary measure (PSS). The impact of the child's health on the caregiver's and family's QOL is measured across the remaining four scales: parental impact-emotional (PE), parental impact-time (PT), family activities (FA), and family cohesion (FC). With the exception of the CH scale, which is analyzed as a categorical variable, all scale measures are transformed to scores ranging from 0 to 100 and are analyzed as continuous variables. Summary measures are standardized with a mean of 50 and standard deviation of 10 to reflect general US population norms for children.

### Child Productivity

The child's school absenteeism and productivity were assessed through question items including: absenteeism in the previous year, late arrivals or early departures from school, missed school-related activities, rescue inhaler utilization at school, and visits to the health office or school nurse because of asthma symptoms.

### Caregiver Work Productivity

A disease-specific version of the Work Productivity and Activity Impairment (WPAI) Questionnaire was used to measure the impact of the child's asthma on the caregiver's productivity [13]. This instrument has been modified in a number of disease areas to assess disease-specific work productivity reductions, rather than general work productivity reductions not necessarily associated with a specific condition [14]. Additionally, this instrument has been modified for use among caregivers [15].

For this study, the instrument was modified to assess impairment that the caregiver attributed to the child's asthma. The WPAI captured the work time absent, impairment while working (presenteeism), overall work productivity impairment, and regular daily activity (eg, work around the house, shopping, studying, exercising) assessed in the previous 7 days.

### Data Analysis

The demographics of caregivers of children with uncontrolled versus controlled asthma were compared with respect to their gender, age, race/ethnicity, and geographical region. Children with uncontrolled versus controlled asthma were also compared on their gender, age and comorbid conditions. HRQOL and productivity differences between children with uncontrolled and controlled asthma and their respective caregivers were analyzed. Differences in means were evaluated using the two-tailed t test procedure, and differences in proportions were evaluated using Fisher's exact test. Because the CH scale in the CHQ-PF28 was measured as an ordinal variable, the Cochran-Armitage test for trend was used to assess differences between the groups. Multiple comparison adjustment using the Bonferroni procedure was made for the 13 domain measures and two summary scales of the CHQ-PF28, all five child productivity measures assessed and all four measures of the WPAI due to number of hypotheses tested simultaneously for these measures. For each statistical test, the statistical level required to meet significance was adjusted by the number of hypotheses tested in order to raise the criteria for meeting significance. Although we are not aware of any formal evaluations to determine the minimal clinically important differences for the CHQ-PF28, others have suggested that most minimal clinically important differences using QOL instruments are centered around 0.5 standard deviation (SD) [12,16].

Guyatt's responsiveness statistic (RS) [17], calculated as a measure's absolute difference between the uncontrolled and controlled groups divided by the standard deviation of the controlled group, was used to describe the effect size of the CHQ-PF28 physical summary measure (PHS) and psychosocial summary measure (PSS) between the uncontrolled and controlled children. Based on the standard deviation criteria for minimally clinically significant differences in HRQOL, an RS greater than 0.5 was interpreted as a moderate effect size, while a RS greater than 0.8 was interpreted as a large effect size [18].

The difference in reduced work productivity between caregivers of children with uncontrolled versus controlled asthma was used to quantify the cost of reduced work productivity due to uncontrolled asthma. Annual cost calculations assumed 220 eight-hour paid working days per year at an average annual salary of $34,426 (or a compensation rate of $19.56/hour) [19]. Statistical analyses were performed using the SAS statistical package (SAS Version 9.1, SAS Institute, Cary, NC).

## Results

**Figure 1:**
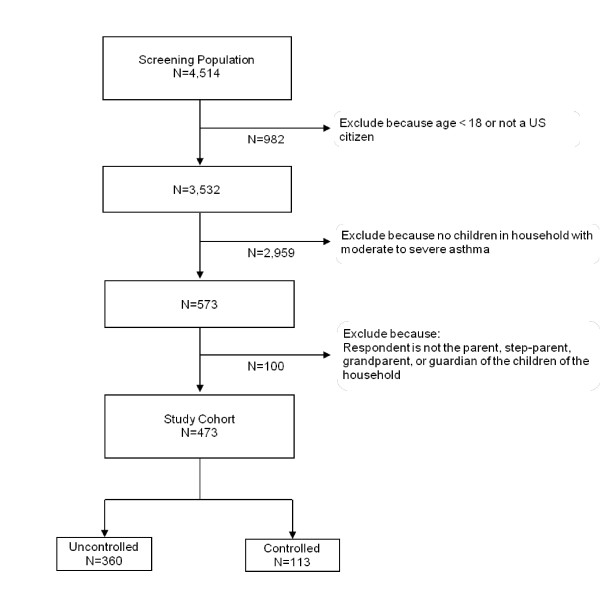
**Study Participation Screening**.

Figure 1 is a flow chart describing the study participation. Invitations to participate in the survey were sent to 16,396 Harris Poll Online members, and 4,514 (25.7%) initiated the survey screener (ie, logged onto the web site) during the 3-week fielding period during June through July of 2007. From this pool of potential participants, participants were queried to identify those who were 18 years of age, a US citizen, and the primary caregiver to a child between the ages of 6 and 12 with asthma within the household. A total of 473 satisfied the study criteria, completed the questionnaire, and were included in this analysis.

### Caregiver Demographics

Of the adult caregivers that met the criteria to participate in this survey, 360 (76%) of 473 had a child classified with uncontrolled asthma. Caregiver age, race/ethnicity, and geographic region distributions were similar between caregivers of children with uncontrolled and controlled asthma (Table 1). Caregivers of children with uncontrolled asthma responding to this survey were more likely to be female. Age and gender of children with asthma did not differ statistically between the two groups. Children with uncontrolled asthma were more likely to have caregiver-reported sinusitis; other co-morbidities were reported with similar frequencies between the groups. Only about one quarter of the children were usually seen by a specialist (allergist, immunologist, or pulmonologist). The majority of the children were usually seen by their pediatrician or general practitioner for their asthma, and this did not vary by control status.

**Table 1:** Characteristics of Caregivers and Children by Asthma Symptom Control

		Uncontrolled	Controlled	*P *value
		N = 360 (%)	N = 113 (%)	
**Caregiver-Specific Characteristics**				

**Gender**				
	Male	83 (23.1)	39 (34.5)	0.0190
	Female	277 (76.9)	74 (65.5)	

**Age**				
	Mean age (SD)	40.4 (9)	41.5 (8)	0.2428

**Race/ethnicity**				
	African American	23 (6.4)	7 (6.2)	0.4282
	Asian/Pacific Islander	2 (0.6)	2 (1.8)	
	Caucasian	312 (86.7)	94 (83.2)	
	Hispanic	9 (2.5)	3 (2.7)	
	Native American	5 (1.4)	2 (1.8)	
	Other	7 (1.9)	2 (1.8)	
	Decline to answer	2 (0.6)	3 (2.7)	

**Region**				
	South	144 (40.0)	39 (34.5)	0.6104
	West	63 (17.5)	18 (15.9)	
	Northeast	65 (18.1)	23 (20.4)	
	Midwest	88 (24.4)	33 (29.2)	

**Child-Specific Characteristics**				

**Gender**				
	Male	216 (60)	78 (69)	0.0956
	Female	144 (40)	35 (31)	

**Age**				
	Mean age (SD)	9.1 (2)	9.1 (2.1)	0.2428

**Type of Physician**				
	Pediatrician	198 (55)	56 (49.6)	
	Family practitioner/general practitioner/internist	63 (17.5)	20 (17.7)	0.82
	Allergist	55 (15.3)	21 (18.6)	
	Immunologist	4 (1.1)	1 (0.9)	
	Pulmonologist	40 (11.1)	15 (13.3)	

**Comorbid Conditions**				
	Eczema or atopic dermatitis	91 (25.3)	26 (23)	0.7081
	Hay fever (seasonal allergic rhinitis)	157 (43.6)	39 (34.5)	0.1006
	Rhinitis	35 (9.7)	10 (8.8)	0.8562
	Sinusitis	87 (24.2)	13 (11.5)	0.0036
	Allergies	265 (73.6)	83 (73.5)	1.0000
	Gastroesophageal reflux disease	37 (10.3)	6 (5.3)	0.1337
	None of these	58 (16.1)	17 (15)	0.8830

Out of the four criteria used to identify children with uncontrolled asthma (Table 2), 81.4% would have qualified for the uncontrolled asthma category based on their night awakenings alone. More than half of the children would have met the criteria for uncontrolled asthma based solely on their activity limitation and nearly half based solely on their daytime symptoms. Caregivers reported that nearly 59.7% of children with uncontrolled asthma met at least two criteria for uncontrolled asthma and one fifth met at least three of the four criteria.

**Table 2:** Criteria for Meeting the Definition of Uncontrolled Asthma

	Uncontrolled Asthma
	N = 360
**Criteria**	**n (%)**

Daytime symptoms	157 (43.6)
Night awakenings	293 (81.4)
Short-acting beta agonist utilization	36 (10.0)
Activity limitation	185 (51.4)

2 or more criteria	215 (59.7)

3 or more criteria	75 (20.8)

All 4 criteria	21 (5.8)

### Health-related Quality of Life

**Figure 2:**
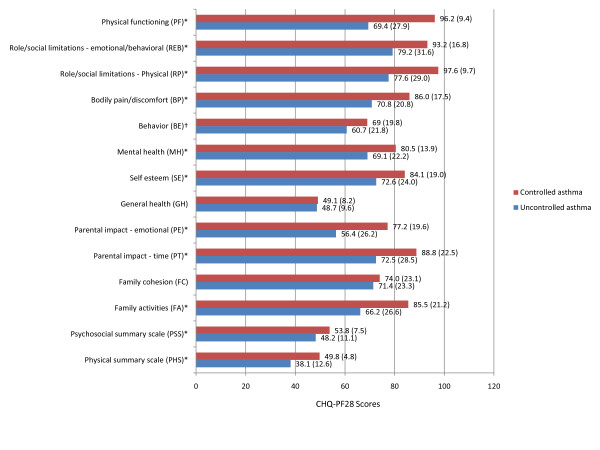
**CHQ-PF28 Scores by Asthma Symptom Control**. All values are displayed as mean (SD). ^‡^*P *≤ 0.005. ^§^*P *≤ 0.0001

Among the nine domains evaluating the HRQOL of the child, children with uncontrolled asthma had significantly worse CHQ-PF28 measures across seven (PF, REB, RP, BP, BE, MH, and SE) when compared with children with controlled asthma (Figure 2). No significant difference was observed in the remaining two scales of change in health and general health. Significant differences were observed in both summary measures; lower mean physical and psychosocial summary measure scores were observed for children with uncontrolled asthma as compared to those whose asthma was controlled by 11.7 points (SE = 1.2, RS = 2.4) and 5.6 points (SE = 1.1, RS = 0.7), respectively (Figure 2). Standardized mean summary scores for children with controlled asthma were within the expected range of population norms (mean = 50, SD = 10) and were within the range for most domains of those scores reported for a large sample of children with no conditions. The mean physical summary scale score for children with uncontrolled asthma was greater than one standard deviation below expected population norms.

A similar impact was observed in the QOL of the child's caregiver and family. Significantly lower scores were observed across three of the four caregiver and family QOL measures (Figure 2). Relative to the mean response of caregivers of children with controlled asthma, the mean response of caregivers of children with uncontrolled asthma was lower across the parental impact-emotional, parental impact-time and family activities scales (all *P *< 0.0001), with significant differences of 26.9%, 18.4%, and 22.6%, respectively. The 3.5% (2.6 points) lower family cohesion scale score observed in the uncontrolled asthma group was not large enough to conclude that an association existed between family cohesion and asthma control status.

### Child Productivity

**Figure 3:**
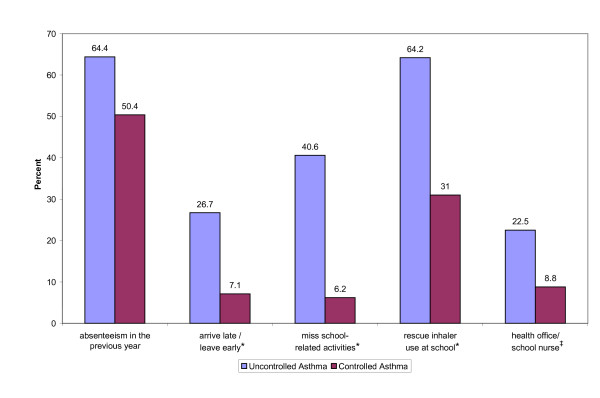
**School Related Measures by Asthma Control**. *P < 0.0001. ^‡^*P *≤ 0.005

Approximately half (50.4%) of the caregivers of children with controlled asthma reported that their child had missed a day of school due to asthma in the past year, while 64.4% of caregivers of children with uncontrolled asthma reported asthma-related absenteeism. On average, children with uncontrolled asthma were reported to miss significantly more days of school (5.5 days, SD = 7.7) than children with controlled asthma (2.2 days, SD = 3.7). Furthermore, compared to the caregivers of children with controlled asthma, a significantly greater percentage of caregivers of children with uncontrolled asthma reported that their child arrived late or departed early from school, missed school-related activities, used a rescue inhaler at school, and visited the health office or school nurse because of asthma symptoms (Figure 3).

### Caregiver Productivity

**Figure 4:**
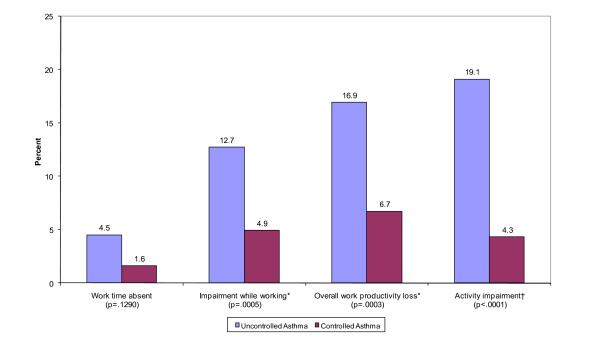
**Mean WPAI Scores for Employed Caregivers by Asthma Control**. *P < 0.0001. ^†^*P *≤ 0.005. Note: Analysis only includes caregivers reporting employment (n = 246 uncontrolled, n = 80 controlled)

No significant difference was observed in the employment status of caregivers of children with uncontrolled versus controlled asthma: 31.7% vs. 29.2% unemployed, respectively. Restricting the analysis to caregivers of children with controlled and uncontrolled asthma who reported employment (n = 246 [68.3%] and n = 80 [70.8%], respectively), caregivers of children with uncontrolled asthma reported a significantly greater work productivity impairment due to the child's asthma across three of the four WPAI measures (Figure 4). On average, these employed caregivers of children with uncontrolled asthma reported nearly three times more work time absent than that reported by caregivers of children with controlled asthma, but due to the reduced sample size in this analysis, especially within the control group, this difference was not large enough to allow a conclusion of statistical significance. Productivity while working was significantly reduced by 12.7% among caregivers of children with uncontrolled asthma versus 4.9% among caregivers of children with controlled asthma. Caregivers of children with uncontrolled asthma also reported a significant work productivity impairment that was 10.2% greater than the impairment reported by caregivers of children with controlled asthma, representing 4.1 hours of additional productivity loss per 40-hour work-week. Furthermore, regular daily activity impairment due to the child's asthma was significantly greater in caregivers of children with uncontrolled asthma.

## Discussion

It has been shown that asthma can have a profound impact on children. Uncontrolled asthma symptoms not only affect children physically but can impair them socially, emotionally, and educationally. However, the impact of asthma in children extends to their caregivers and families, who face the burden of care and impact on lifestyle. Achieving optimal asthma control can reduce the impact of symptoms on the daily functioning of the child in addition to the caregivers and other family members.

By surveying caregivers of children with moderate to severe asthma, we evaluated the impact of uncontrolled asthma on children and their caregivers among a random sample from a general registry of Internet users representative of the US adult population. In this study, the frequency and severity of symptoms were sufficient that three quarters (76%) could be classified as uncontrolled. Given that uncontrolled asthma is reported at approximately 60% in general practice populations [20,21], the high rate of uncontrolled symptoms among children with moderate to severe asthma in this study is not completely unexpected and highlights the under-management of asthma in the pediatric population [11]. Although studies such as this one indicate that symptom control is achieved far less optimally in real world practice settings, it has been shown that asthma control can be achieved and maintained in the majority of patients [22].

Children with uncontrolled asthma had significantly lower HRQOL scores across seven of nine CHQ-PF28 domains relating to the physical, emotional, and social well-being of the child, demonstrating the extent of the effect of uncontrolled symptoms on the child. Within the school experience, children with uncontrolled asthma missed a significantly greater number of school days than their controlled counterparts. Even when children were present within school, results suggest that children whose asthma disease state is not well controlled miss more classes due to arriving late, leaving early, and visiting the health office and school nurse, and miss more school-related activities compared to children with controlled asthma. Given the impact asthma has on school, creating and utilizing individual asthma action plans within the school and maintaining communication between teachers and caregivers should be considered a part of the child's treatment plan.

Uncontrolled pediatric asthma also had a negative impact on the family and caregivers. Although caring for a child with asthma requires caregiver time, and families of children with controlled asthma must avoid some types of activities, caregivers of children with uncontrolled asthma report even lower HRQOL scores than those reported in controlled asthma, suggesting that uncontrolled asthma exacts an even greater toll on the caregiver and families.

The effects of uncontrolled asthma on the caregiver extend beyond the social and emotional impact. Among employed caregivers, work productivity impairment was significantly greater among parents of children with uncontrolled asthma. Compared with employed caregivers of children with controlled asthma, employed caregivers of children with uncontrolled asthma had an additional 10.2% overall work productivity impairment. This difference amounts to an average cost of $3,511 in estimated annual incremental costs above that seen in employed caregivers of children with controlled asthma. Findings from this study suggest that children with uncontrolled asthma are far more likely to experience asthma-related nighttime awakenings, and it is not at all unlikely that their caregivers too are awakened more often at night. This could be a driving factor in impaired work performance the next day. With decreased overall productivity and the concerns of caring for their child, issues of job security may also be of concern for parents.

A number of studies have highlighted an association between increasing asthma severity in children and reduced quality of life and absenteeism while others have found differing results [23,24]. Some of this discrepancy may be due to inconsistencies in the methods and criteria used to define asthma severity. With the shift from asthma severity to asthma control in the diagnosis and management of asthma, a greater need for measuring and understanding the burden of uncontrolled asthma is essential. This study provides a method for defining asthma control that closely follows criteria outlined in the NAEPP 2007 asthma guidelines [11]. Findings from this study support those reported by others, reflecting that better asthma control is associated with better outcomes [25,26].

[22]This study relied on the information provided by primary caregivers for their children with asthma. The same is true for physicians of pediatric patients, who have to obtain their information regarding symptom control from the caregivers. It is essential for physicians to provide the tools for these caregivers on how to observe their children and monitor their asthma symptoms. Physicians and parents need to communicate and work together to establish control over asthma and monitor closely when this state changes. Physicians can help the caregivers in this process by providing direction through a written action plan.

This research has some limitations. As mentioned previously, the use of an Internet population may limit the ability to generalize the results of this study. Typically, Internet users tend to have higher education and income than the general population among other differences, and the prevalence and impact of uncontrolled asthma may be worse for patients and families with lower income and less access to health care. However, web-based surveys are increasing in popularity as a means of reaching large numbers of patients even in the area of asthma [27], and research evaluating web-based surveys among general research panels against other epidemiologic forms of data collection suggest their comparability [28,29]. The response rate in this study--25%--compares well with other web-based surveys as suggested by the 26.5% median response rate (meaning that half of all surveys get at least a 26.5% response rate) that was reported in a recent white paper written for industry guidance for online survey use [30]. Additionally, it should be pointed out that participation of patients with controlled and uncontrolled asthma would likely not be differentially biased since all respondents were from the same internet pool; care should be taken when generalizing to the broader population.

As with any survey, recall bias may affect interpretation of results. Caregivers of children with uncontrolled asthma may be uniquely aware, and therefore have differing recall, of their child's symptoms and measured outcomes. In addition, this study used a generic HRQOL instrument and a modified version of a productivity instrument to determine asthma-specific impact of disease control on children and caregivers. A generic HRQOL instrument was chosen in part because this survey was completed by caregivers on behalf of their children rather than by direct child assessment and because few disease-specific instruments allow for HRQOL among young children reported by their caregivers. Although the WPAI has been modified and validated in a number of disease areas and for use among caregivers, the caregiver asthma-specific version has not undergone formal validation. The inclusion of children with moderate to severe asthma in this study was based on patterns of medication and utilization reported during the previous 6 months. The use of medications to classify asthma severity can be complicated and is based on assumptions about treatment adequacy that cannot be verified within the current study design. Lastly, based on the NAEPP guidelines, the reporting of interference with normal activities was used to classify asthma control status. However, interference with normal activities is also a strong component of HRQOL and thus we may have influenced the inter-group comparison of HRQOL measures based on the definition of uncontrolled asthma alone.

The algorithm for symptom control used in this study was determined by question responses regarding key symptom control expressions described in the NAEPP 2007 asthma guidelines. However, recruitment for this study began prior to the release of the NAEPP update in August 2007. As such, response options for frequency of rescue inhaler use did not map exactly to the limits set forth in the current guidelines. We chose to use a more conservative measure of rescue inhaler use (ie, a greater frequency of usage) to categorize control, which may have led to some children being misclassified as controlled, thus underestimating the differences between children with uncontrolled and controlled asthma.

## Conclusion

In conclusion, caregivers of children with asthma face many challenges and can also be profoundly impacted by their child's illness. Uncontrolled asthma has a significant impact on the HRQOL and productivity of children and on the QOL and work productivity of their caregivers, and has an impact on their families.

## Abbreviations

BP: bodily pain/discomfort; BE: behavior; CH: change in health; CHQ-PF28: Child Health Questionnaire Parent Form 28; FA: family activities; FC: family cohesion; GH: general health; HRQOL: health-related quality of life; MH: mental health; NAEPP: National Asthma Education and Prevention Program; PE: parental impact-emotional; PF: physical functioning; PHS: physical summary measure; PSS: psychosocial summary measure; PT: parental impact-time; QOL: quality of life; REB: role/social limitations-emotional/behavioral; RB: role/social limitations-physical; RS: Guyatt's responsiveness statistic; SE: self-esteem; also standard error; US: United States; WPAI: Work Productivity and Activity Impairment

## Competing interests

The research presented in this paper was supported by an unrestricted grant from Novartis Pharmaceuticals Corporation (Novartis). BBD, DA and BCC are employed by Cerner LifeSciences, which provides consulting services to the pharmaceutical industry. RM and PS are employees of Novartis and therefore receive compensation from the study sponsor in the form of personal wages and in equity/ownership (e.g., company stock) in the company.

## Authors' contributions

Each author has participated in the concept and design; analysis and interpretation of data; drafting or revising of the manuscript and each author has read and approved the manuscript as submitted. Each author has disclosed any affiliation, financial agreement, or other involvement with any company whose product figures prominently in the submitted manuscript so that the editors can discuss with the affected authors whether to print this information and in what manner.
